# Diagnosis of drug-induced renal tubular toxicity using global gene expression profiles

**DOI:** 10.1186/1479-5876-5-47

**Published:** 2007-10-01

**Authors:** Ying Jiang, David L Gerhold, Daniel J Holder, David J Figueroa, Wendy J Bailey, Ping Guan, Thomas R Skopek, Frank D Sistare, Joseph F Sina

**Affiliations:** 1 Safety Assessment, Merck Research Laboratories, Merck & Co., Inc, 770 Sumneytown Pike, West Point, PA 19486, USA; 2 Biometric Research, Merck Research Laboratories, Merck & Co., Inc, 770 Sumneytown Pike, West Point, PA 19486, USA

## Abstract

Toxicogenomics can measure the expression of thousands of genes to identify changes associated with drug induced toxicities. It is expected that toxicogenomics can be an alternative or complementary approach in preclinical drug safety evaluation to identify or predict drug induced toxicities. One of the major concerns in applying toxicogenomics to diagnose or predict drug induced organ toxicity, is how generalizable the statistical classification model is when derived from small datasets? Here we presented that a diagnosis of kidney proximal tubule toxicity, measured by pathology, can successfully be achieved even with a study design of limited number of training studies or samples. We selected a total of ten kidney toxicants, designed the in life study with multiple dose and multiple time points to cover samples at doses and time points with or without concurrent toxicity. We employed SVM (Support Vector Machine) as the classification algorithm for the toxicogenomic diagnosis of kidney proximal tubule toxicity. Instead of applying cross validation methods, we used an independent testing set by dividing the studies or samples into independent training and testing sets to evaluate the diagnostic performance. We achieved a Sn (sensitivity) = 88% and a Sp (specificity) = 91%. The diagnosis performance underscores the potential application of toxicogenomics in a preclinical lead optimization process of drugs entering into development.

## Background

Drug discovery and development is an expensive and time consuming process. It is estimated that about one third of drug candidates are terminated due to lack of clinical safety or toxicity concerns [1-3]. Identifying drug safety liabilities or predictive biomarkers for drug induced organ damage at or before the preclinical stages of drug development is of great importance to pharmaceutical companies. The ability to make proper go or no go decisions based on safety would greatly reduce the cost of drug development and improve the attrition rate of new chemical entities (NCE). Preclinical drug safety evaluation, at this time, mainly relies on complex histopathological or clinical pathological analysis. These traditional approaches have proven to be highly successful but may fail to detect benign or prodromal stages of toxicity. Gene expression profiling stands as a complementary or possibly alternative molecular diagnosis approach. Transcriptional profiling has the promise of being able to detect toxicity objectively, accurately and earlier, while requiring considerably less time and resources. Gene expression changes from preclinical studies associated with toxicity may also assist with our understanding of the mechanism of certain drug induced toxicities [4].

The kidney is a major organ for filtration, secretion, re-absorption and ultimately excretion of drugs or drug metabolites. As a consequence of its primary function, the kidney is especially vulnerable to toxic insults by various drugs or xenobiotics, and thus nephrotoxicity is one of the major concerns in preclinical safety evaluation. Despite the morphological complexity of the kidney, the renal tubular epithelial cells stand out as one of the most sensitive components in the kidney and are thus highly susceptible to damage. Drug induced tubular damage has been well documented and studied extensively [5]. Molecular methods using microarray gene expression data have been attempted to predict and diagnose preclinical renal tubular toxicity. Fielden and colleagues [6] used a strategy designed to assess predicative gene expression endpoints at early time points proceeding the onset of any signs for renal tubular pathology. They achieved a sensitivity of 76% which is much better than traditional approaches which often have no significant prediction values. In a separate study designed to assess the expression profiling end points in matching the histopathological diagnosis of concurrent renal tubular toxicity, the performance was improved and a sensitivity of 82% was achieved [7].

The success of statistically modeling microarray gene expression data to diagnose or predict renal toxicity is often constrained by a limited number of samples in combination with a large number of features (genes) to be monitored. This common conundrum is typically the case with most "omics" studies and has been referred to as "the curses of sample sparsity and feature dimensionality" [8] which often lead to an over-fitted, non generalizable statistical model or poor prediction performance. The general approach to overcome or avoid such problems, is to design a representative training set (random sampling), and estimate the model performance with an independent testing data set. However, for a toxicogenomics prediction of future onset of toxicity, it is very difficult to have a study design with enough coverage of compounds, doses and time-points to cover all possible mechanistic prodromal signals due to the biological complexities of the whole compound space. A likely exception to this is the diagnosis of drug induced acute organ toxicity. It is assumed that there are defined molecular manifestations or transcriptional changes that occur when a very specific toxicity occurs. Thus we hypothesized that a study involving multiple doses and time points for a small number of defined compounds with different pharmacological mechanisms will allow uncommon pharmacological responses to be neutralized and diagnostic gene expression changes associated with toxicity to be defined. Such gene expression profiling end points have shown to provide accurate diagnostic information as demonstrated by the two studies referenced above and the diagnostic exercise gave better performance than the predicative exercise, although the performances were evaluated by cross validation [6,7]. Other carefully designed profiling studies with limited number of samples have also been attempted in preclinical drug development with certain success [9-12].

The support vector machine (SVM) algorithm [13] is one of the most powerful supervised learning algorithms in microarray or gene expression profiling data analysis [14,15]. Used as a "one over all" binary classifier to perform multi-class cancer diagnosis, SVM has been shown to outperform other classification methods consistently [16]. In this report, by applying a binary SVM classification algorithm, to a well designed microarray gene expression dataset, using an independent testing dataset to evaluate classification performance, we report much better diagnosis of kidney tubular toxicity in rats.

## Methods

### Animal husbandry

Two groups of independent in-life studies were carried out by Merck and Charles River Laboratories (See Table 1). For both Charles River Laboratories and Merck studies, male Sprague-Dawley (SD) rats (320–370 g) approximately 11-weeks-old, from Charles River Laboratories were used. The animals were individually housed in metabolic cages. Certified Rodent Diet #PMI 5002 was provided at 22 grams of feed daily at the beginning of acclimation through study termination. In the event of feed remaining at the end of day, all remaining feed was removed before providing the next daily feed allocation. Filtered tap water was provided ad libitum. The rats were kept at a controlled temperature of 61–79 F and at a humidity of 30–70%. A 12:12 hour light:dark cycle was maintained in the animal room.

**Table 1:** In vivo compound treatments used in training and testing

**Compound**	**Class**	**Conducted by**	**Dose (mpk)**	**Necropsy day**	**Vehicle – Route**
Cisplatin	DNA – alkylator	Merck	0.5	3, 8	0.9% (w/v) sodium chloride – IP
			3.5	3, 8	
			7	3, 8	
Cyclosporin A	Calcineurin inhibitor	Merck	6	3, 9, 15	olive oil – SC
			30	3, 9, 15	
			60	3, 9, 15	
Gentamycin	Antibiotic	Merck	20	3, 9, 15	0.9% (w/v) sodium chloride – IP
			80	3, 9, 15	
			240	3, 9, 12	
Sodium Fluoride	Environmental toxin	Merck	35	3, 8, 12	Water – PO
			75	3, 8, 12	
Merck X	Antibiotic	Merck	75	3, 8, 14	0.5%saline – IV
			150	3, 8, 14	
			225	3, 8	
Allopurinol	Xanthine oxidase inhibitor	Charles River	6	3	corn oil – IP
			30	3	
			100	3, 7, 14	
D-serine	Serine analog	Charles River	750	3, 14	water – IP
Hexachloro 1,3, butadiene	Synthetic toxin	Charles River	7.5	3	corn oil – IP
			40	3, 14	
			100	3	
Puromycin	Antibiotic	Charles River	5	3	0.9% (w/v) sodium chloride – IP
			20	3, 7, 14	
			60	3, 7	
Tobramycin	Antibiotic	Charles River	6	3	0.9% (w/v) sodium chloride – IP
			30	14	
			60	3, 14	

Male Sprague-Dawley rats were treated daily with the listed compounds except D-serine which was given as a single dose once on day 0. Each dose group includes 4 or 5 rats. Animals were terminated at the end of study. Terminal or interim necropsy were performed 24 hours post dosing. Kidney expression profiles were obtained for each necropsy day (when kidney samples were harvested). Appropriate dosing routes were applied: PO – Oral Garvage, IV – intravenous, SC – subcutaneous.

### Test articles and study design

Test articles were suspended or dissolved in respective vehicles (see Table 1) and dosed via the individual routes. Most of the test articles involve multiple doses and repeat daily dosing, except D-serine which was given as a single dosing. The studies were about two weeks in duration with interim necropsy or sampling (Table 1).

### Tissue collection and histopathology

For Charles River studies, animals were humanely euthanized by group via anesthesia with carbon dioxide to effect followed by exsanguination and submitted for a complete necropsy examination (defined as examination of the external surface of the body; all orifices; and the cranial, thoracic, and abdominal cavities, and their contents). For histopathological evaluation, a kidney from each animal was examined in situ, dissected free, and fixed in 10% neutral buffered formalin. Histopathology was performed on kidney from all animals (except animals found dead). Fixed tissues were trimmed, embedded, and sectioned. Slides were stained with hematoxylin and eosin. To collect kidney samples for RNA profiling, the second kidney from each animal was dissected free and placed in tubes containing RNAlater™ (Qiagen). The tubes were allowed to remain at room temperature for a minimum of 30 minutes (maximum of 2 hours) before freezing at -20°C.

Similarly for Merck conducted studies, rats were anesthetized under isoflurane, bled via the vena cava, exsanguinated and necropsied. Kidneys were subject to preparation for histopathology examination and toxicogenomics study as described above.

### RNA extraction and expression profiling

RNA extraction, expression profiling, data processing and quality control were performed as previously described (De Souza et al. 2006). Briefly, RNA was extracted from tissues using a combination of TRIzol RNA extraction (Invitrogen, Carlsbad, CA) with RNeasy RNA extraction kit (Qiagen, Valencia, CA). Expression profiling was carried out using Rosetta custom arrays consisting of 22.5 K 60 mer oligonucleotides (plus control sequences) representing rat genes, Rat 2.25 K chip. The arrays were synthesized using The Agilient inkjet printing method. Two Cy3- and Cy5- two color reverse hybridization was applied. Individual samples were hybridized against a pool of RNA from time matched (concurrent) control animals. The ratio of individual animal expression to control pool was used for all data analysis. All hybridizations were performed in duplicate, with fluor reversal (Cy3 or Cy5) in the second hybridization. The resultant fluo-reversed pairs were combined to give a single ratio measurement for each gene of each sample.

### Support Vector Machine (SVM)

SVM light is from Thorsten Joachims [17]. Briefly the samples were naturally divided into independent training and testing sets of Merck studies and Charles River Laboratories studies respectively. LOOCV (Leave One Out Cross Validation) was performed for optimization.

### Spotfire

Spotfire™ is a type of data analysis and visualization software licensed from business intelligence company TIBCO [18]. Spotfire was used here to produce a heatmap to illustrate the correlation of gene expression with SVM prediction.

## Results

The paradigm used for the study design or data analysis assumes that any test compound can be toxic at a given dose and time. A drug is safe as long as margins which fall short of its toxicity can be established. Therefore, preclinical drug safety assessment is more concerned with diagnosed drug toxicity relative its effective dose and duration of dosing. A study design of multiple doses with different time points covering toxic dose/time and non toxic dose/time enables the differentiation of gene expression changes associated with toxicity from those due to pharmacology and can potentially define safety margins. If the study design is expanded to incorporate several structurally different compounds which induce the same toxicity by pathology, the different pharmacological effects reflected in the gene expression can be further diminished or neutralized in the analysis (by cancelling each other out), while at the same time gene expression changes associated with the defined common toxicity are qualified for the purpose of diagnosis of such toxicity. As an alternative to the conventional analysis which would require involving large numbers of studies or compounds in the training set, the above reasoning underscores the importance of this type of focused expression profiling design for diagnosis drug induced toxicity.

As described in materials and methods, a total of nine kidney proximal tubule toxicants and one glomerular toxicant were selected for the study. All of them are known for inducing kidney toxicities, mainly proximal tubule toxicities identified as necrosis/degeneration by pathology. Puromycin and Tobramycin are known to cause a combination of glomerular and proximal tubule toxicity [7]. The toxicants were chosen based on their known kidney toxicity profile or availability. The in vivo studies were divided into two groups and conducted by either Merck or Charles River Laboratory. Multiple dose levels and repeat dosing were designed except for D-serine with a single dose (Table 1). Kidney tissues were collected at necropsy and subjected to microarray gene expression study and histopathology analysis (see methods). Interim necropsy was performed so to obtain data from multiple time points for most studies.

A standard approach to the pathological evaluation was employed. Significant histopathological finding for PT toxicity were summarized animal by animal. Merck studies are shown in supplement data, Charles River studies are shown in Table 2. A grade was assigned which qualitatively represent the severity of the toxicity. The grading was agreed upon by consensus of a peer review committee of pathologists. Grade 0 represents no histopathology, or non toxic, while Grades 1 or higher represent toxicity observed by histopathology.

**Table 2:** Charles River Laboratories study

A_id	C.Dose.Day	H_score	B_class	A_id	C.Dose.Day	H_score	B_class
1	All.006.03	0	-1	65	Pur.075.03	0	-1
2	All.006.03	0	-1	66	Pur.075.03	0	-1
3	All.006.03	0	-1	67	Pur.075.03	0	-1
4	All.006.03	0	-1	68	Pur.075.03	0	-1
5	All.030.03	0	-1	69	Pur.075.07	3	1
6	All.030.03	0	-1	70	Pur.075.07	2	1
7	All.030.03	0	-1	71	Pur.075.07	0	-1
8	All.030.03	0	-1	72	Pur.075.07	0	-1
9	All.100.03	2	1	73	Tob.006.03	0	-1
10	All.100.03	2	1	74	Tob.006.03	0	-1
11	All.100.03	2	1	75	Tob.006.03	0	-1
12	All.100.03	2	1	76	Tob.006.03	0	-1
13	All.100.03	1	1	77	Tob.030.14	1	1
14	All.100.03	1	1	78	Tob.030.14	0	-1
15	All.100.03	1	1	79	Tob.030.14	0	-1
16	All.100.03	1	1	80	Tob.030.14	0	-1
17	All.100.07	2	1	81	Tob.060.03	0	-1
18	All.100.07	2	1	82	Tob.060.03	0	-1
19	All.100.07	1	1	83	Tob.060.03	0	-1
20	All.100.07	1	1	84	Tob.060.03	0	-1
21	All.100.14	2	1	85	Tob.060.14	2	1
22	All.100.14	2	1	86	Tob.060.14	2	1
23	All.100.14	2	1	87	Tob.060.14	2	1
24	All.100.14	1	1	88	Tob.060.14	2	1
25	D-S.750.03	4	1	89	Veh.000.03	1	1
26	D-S.750.03	4	1	90	Veh.000.03	0	-1
27	D-S.750.03	4	1	91	Veh.000.03	0	-1
28	D-S.750.03	0	-1	92	Veh.000.03	0	-1
29	D-S.750.14	2	1	93	Veh.000.03	0	-1
30	D-S.750.14	2	1	94	Veh.000.03	0	-1
31	D-S.750.14	2	1	95	Veh.000.03	0	-1
32	HCB.007.5.03	0	-1	96	Veh.000.03	0	-1
33	HCB.007.5.03	0	-1	97	Veh.000.03	0	-1
34	HCB.007.5.03	0	-1	98	Veh.000.03	0	-1
35	HCB.040.03	1	1	99	Veh.000.03	0	-1
36	HCB.040.03	0	-1	100	Veh.000.03	0	-1
37	HCB.040.03	0	-1	101	Veh.000.03	0	-1
38	HCB.040.14	2	1	102	Veh.000.03	0	-1
39	HCB.040.14	2	1	103	Veh.000.03	0	-1
40	HCB.040.14	2	1	104	Veh.000.03	0	-1
41	HCB.040.14	2	1	105	Veh.000.03	0	-1
42	HCB.100.03	4	1	106	Veh.000.03	0	-1
43	HCB.100.03	4	1	107	Veh.000.03	0	-1
44	HCB.100.03	3	1	108	Veh.000.03	0	-1
45	HCB.100.03	3	1	109	Veh.000.07	1	1
46	Pur.005.03	0	-1	110	Veh.000.07	0	-1
47	Pur.005.03	0	-1	111	Veh.000.07	0	-1
48	Pur.005.03	0	-1	112	Veh.000.07	0	-1
49	Pur.005.03	0	-1	113	Veh.000.07	0	-1
50	Pur.020.03	2	1	114	Veh.000.07	0	-1
51	Pur.020.03	0	-1	115	Veh.000.07	0	-1
52	Pur.020.03	0	-1	116	Veh.000.07	0	-1
53	Pur.020.03	0	-1	117	Veh.000.14	1	1
54	Pur.020.03	0	-1	118	Veh.000.14	1	1
55	Pur.020.03	0	-1	119	Veh.000.14	0	-1
56	Pur.020.03	0	-1	120	Veh.000.14	0	-1
57	Pur.020.03	0	-1	121	Veh.000.14	0	-1
58	Pur.020.07	0	-1	122	Veh.000.14	0	-1
59	Pur.020.07	0	-1	123	Veh.000.14	0	-1
60	Pur.020.07	0	-1	124	Veh.000.14	0	-1
61	Pur.020.14	3	1	125	Veh.000.14	0	-1
62	Pur.020.14	3	1	126	Veh.000.14	0	-1
63	Pur.020.14	3	1	127	Veh.000.14	0	-1
64	Pur.020.14	2	1	128	Veh.000.14	0	-1

Charles River Laboratories samples with histopathological grade and binary class label for SVM classification. The columns are: A_id, the animal identification number; C.Dose.Day, Compound.Dose.Day; H_score, histopathology grade; B_class, the designated SVM class label for binary classification training or testing. The compounds are: All (Allopurinol), D-s (D-serine), HCB (Hexachloro 1,3, butadiene), Pur (Puromycin), Tob (Tobramycin) and Veh (Vehicle control). The designated SVM binary class label was assigned based on the pathology grade. 1–5 labeled as 1, the positive class and 0 labeled as -1, the negative class.

Supervised learning algorithm, SVM, was employed as the binary classification algorithm for its reported better performance as discussed in introduction. Histopathology observations were used as the anchor for the SVM training and testing for the kidney proximal tubule toxicity classification. Histopathology scores of 1 or higher for tubular toxicity were viewed as toxicity class and labeled as +1, while vehicle controls or treated samples with histopathology score of 0 were assigned in negative class and labeled as -1. Such class labels were used to supervise the SVM learning during SVM training and to measure the SVM model performance while in testing. SVM class designations for samples from Charles River Laboratories are listed in table 2 and the Merck samples are listed in additional file 1.

In brief, there were a total of 250 Merck samples. Forty-one of these samples have no histopathology data available (not performed), leaving 209 samples with histopathology, to be included in the data analysis. 96 of the 209 Merck samples had tubular toxicity (pathology grade 1 to 5). In the studies conducted by Charles River Laboratories, a pathology evaluation was carried out on 128 samples, and 45 out of 128 had tubular toxicity indicated by pathology including 4 vehicle controls. The tubular pathology in the vehicle controls was considered non drug treatment related and was potentially misclassifications. These four vehicle samples could be excluded from analysis. Microarray gene expression data for all the samples was generated using the Agilient rat 22.5 K chip. The log ratio of the gene expression for the ratio of treated versus control pool was used as the feature values in SVM training and testing described below.

Since the number of Merck samples is more than that of Charles River samples, Merck samples were used for training the SVM algorithm. Charles River studies were used as the independent testing set. When training SVM with Merck samples, linear kernel function (linear SVM) was selected and leave one out cross validation (LOOCV) was performed. For performance testing with the independent Charles River study samples, a positive SVM score indicates predicted positive or toxic and negative SVM score indicates predicted non toxic (SVM predicted class label shown in supplement data and Table 2, SVM scores not shown). The testing accuracy of 87% was achieved with Sn = 81%, Sp = 91% initially, see Table 3. When we inspect the miss classified samples in the Charles River testing set, we noticed four of the false negative predictions are the four vehicle controls with the pathology grade 1. Concerned with potential miss calls by histopathology and considering the fact that they were not drug treatment related even if they were correct calls, we decided to exclude those four vehicle controls and recalculate the performance of the SVM classification. This time The Sn is 88% and Sp is 91%. This is the best performance achieved from an independent testing as opposed to a cross validation.

**Table 3:** Testing results

	**Tubular Toxicity Test**			
	
	**with 4 veh positives**		**without 4 veh positives**	
	
	**TRUE**	**FALSE**	**TRUE**	**FALSE**
**Positive**	38	7	38	7
**Negative**	74	9	74	5
				
**Sn**	81% = 38/(38 + 9)		88% = 38/(38 + 5)	
**Sp**	91% = 74/(74 + 7)		91% = 74/(74 + 7)	
**Positive Prediction**	84% = 38/(38 + 7)		84% = 38/(38 + 7)	
**Negative Prediction**	89% = 74/(74 + 9)		94% = 74/(74 + 5)	

Linear SVM model was built using Merck studies as training set. Using Charles River Laboratories studies as testing set, sensitivity and specificity as well as Positive prediction of kidney proximal tubule toxicity classification were obtained.

**Figure 1:**
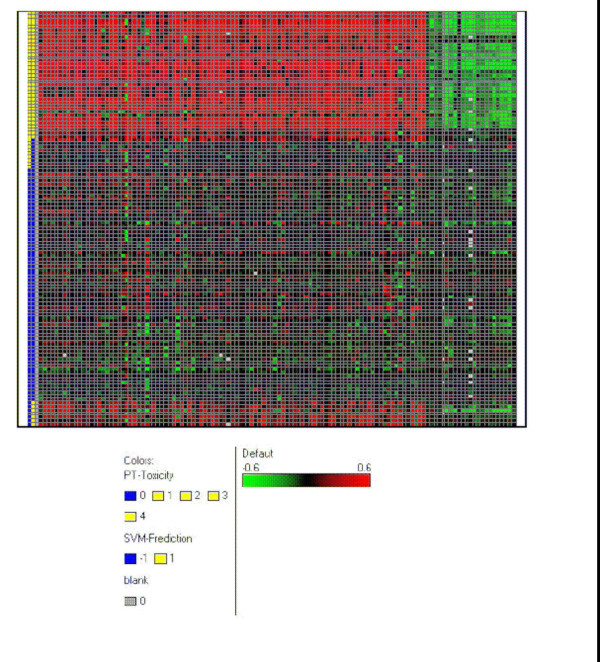
**Heatmap to illustrate the kidney proximal tuble toxicity classification by SVM**. Top ranked 100 up regulated genes (positive weighted) and 25 down regulated genes (down regulated) by linear SVM were used to correlate with the kidney proximal tubule toxicity and SVM predicted class label. The first column is PT histopathology grade. The second column is the SVM predicted class label: -1 is predicted non toxic and 1 is predicted toxic. After a Blank column for separation, the rest columns are the selected top ranked genes in logratios. The rows in the heatmap represent samples.

The linear SVM algorithm employed here used all the genes or features on the chip to perform the kidney proximal tubule toxicity classification, although genes are used independently and uniquely as to their importance. SVM algorithm is generally known to work as a black box without much interpretability of how individual feature contributes to the SVM model performance; however the linear SVM does assign a weight or coefficient [19] to each of the features. Such feature weight when considered together with the feature value can be used to estimate the relative significance of individual features in linear SVM classification. Here we first parsed out the gene weights from the linear SVM model, then we calculate the product of the gene weight and the gene fold change (individual sample versus control pool, see Materials and Methods) for each gene and each sample with kidney proximal tubule toxicity. The averaged product value obtained over all toxic samples in the training sample set is used to rank all the genes. We took the top ranked 100 positive weighted and top ranked 25 negative weighted genes to generate a heatmap using spotfire™ (Methods). Figure 1 shows the heatmap which illustrates the top SVM classifier genes against samples with and without kidney proximal tubule toxicity. The gene expression changes are consistent with the SVM predicted class labels. Very little gene expression changes were seen with the false negative samples while gene expression changes did appear for false positive samples (the samples are shown as non matching colors in the first two columns indicating SVM mis-classification, false negative or false positive).

The positive prediction and negative prediction values by this SVM classification model are also listed in Table 3. It was noted that some positive Puromycin and Tobramycin samples were falsely identified as negatives (false negatives) in our study (Table 4). Similar findings have been noted by others [7], and it has been speculated that their associated glomerular toxicity may have complicated and confounded the diagnosis of their PT toxicity.

**Table 4:** The 16 mis-classified samples

**A_id**	**Animal**	**Treatment**	**Cpd.Dose.Day**	**H_score**	**B_class**	**SVM_value**	**Class_predicted**	**TRUE/FALSE**
84	2021	Tobramycin	Tob.060.03	0	-1	0.053417599	1	FALSE
80	2017	Tobramycin	Tob.030.14	0	-1	0.020981321	1	FALSE
36	2071	HCB	HCB.040.03	0	-1	0.3853432	1	FALSE
5	2117	Allopurinol	All.030.03	0	-1	0.4184979	1	FALSE
6	2118	Allopurinol	All.030.03	0	-1	0.33539873	1	FALSE
7	2119	Allopurinol	All.030.03	0	-1	0.079393616	1	FALSE
8	2120	Allopurinol	All.030.03	0	-1	0.40372499	1	FALSE
17	2142	Allopurinol	All.100.07	1	1	-0.33466752	-1	FALSE
19	2143	Allopurinol	All.100.07	1	1	-0.25854402	-1	FALSE
104	2408	Vehicle	Veh.000.03	1	1	-1.4422447	-1	FALSE
121	2413	Vehicle	Veh.000.14	1	1	-1.277754	-1	FALSE
128	1940	Vehicle	Veh.000.14	1	1	-1.2135719	-1	FALSE
116	1936	Vehicle	Veh.000.07	1	1	-1.1563262	-1	FALSE
54	1962	Puromycin	Pur.020.03	2	1	-1.0786993	-1	FALSE
63	1971	Puromycin	Pur.020.14	2	1	-1.3810734	-1	FALSE
61	1969	Puromycin	Pur.020.14	3	1	-0.056223804	-1	FALSE

The mis-classified samples by SVM in Charles River Laboratories studies are listed here. Columns are: A_id, animal identification; Treatment, compound; Cpd.Dose.Day, compound.dose.day; H_score, histopathology grade; B_class, designated SVM class label for testing; SVM value, the prediction value from SVM model (>0 indicates positive class and <0 indicates negative class); class_predicted, predicted class label; Prediction, prediction true or false.

By reversing the data sets and use the Charles River study for training, the Merck samples for testing, we can achieve an accuracy of 81%, which is similar to but less than the 87% accuracy achieved above. This is most likely due to the fact that there are almost twice as many Merck samples as Charles River samples. Knowing the acceptable performance of the linear SVM model by independent testing, as a common practice in data mining to ensure optimal training, we can actually train the linear SVM model with both Merck and Charles River samples for future diagnosis of kidney proximal tubule toxicity.

## Discussion

Using toxicogenomics data to diagnose histopathology of kidney proximal tubule toxicity has usually achieved accuracy in the eighties. Thukral SK et al. [7] attempted to predict prognosis of kidney proximal tubule toxicity by diagnosing subtypes or subtype combination of kidney proximal tubule histopathology. A sensitivity of 82% was achieved. This is a very good performance for toxicity subtype prediction even though only one compound was used for testing. Similarly anchored with histopathology, in this report, we grouped all proximal tubule toxicity as one positive class including grade 1 for the slightest pathology. We reported better performance in diagnosis of concurrent kidney proximal tubule toxicity using genomics with sensitivity of 88% and specificity of 91%. We did so using independent testing dataset consists of 5 different compounds, which is a better estimate of the true performance. The study design only involved 10 toxicants, therefore the success in this exercise also implied that sample sparsity [8], which often complicates most genomics or microarray data analysis may not be as big a problem in diagnosis of concurrent toxicities as discussed earlier, it also implies that such focused genes expression profiling experiment design may be generally applicable to diagnose drug induced organ toxicities. However, also as discussed earlier, the same can not be said for toxicogenomics prediction of later or future onset of drug induced toxicities. Such predictive toxicogenomics requires more representative sample coverage of diverse prodromal mechanisms leading to toxicity, when sample sparsity is actually going to be a difficult hurdle to overcome.

On the other hand, for diagnosis of drug induced concurrent toxicity, in this report, the SVM model built with thousands of genes as features gave highly desirable performance, without requesting the understanding of how genes in the SVM model contribute to the classification. If interpretability of the diagnosis is of concern, feature selection algorithms could be applied to identify the more important genes or features for the classification or diagnosis of the toxicity of interest.

An additional exercise (not shown) using random half of the genes on the microarray to do the classification, similar performance could be achieved. Thus reducing the number of genes in the model does not really affect the classification performance as much. This implies that there is rich and maybe redundant classification information in the gene expression profiles. Such rich toxicogenomics diagnosis information, in turn, confirms that the study design of small number of compounds representing different pharmacology is a working design for diagnosis of concurrent toxicity (identified by histopathology).

Our effort here was primarily to apply SVM and microarray gene expression profiles in diagnosis of concurrent kidney proximal tubule pathology. It could be potentially applied to diagnosis of other well defined drug induced toxicity. With the cost of profiling experiments going down, such toxicogenomics approach could be applied early in lead optimization or it would even be integrated into preclinical drug safety assessment processes, so to reduce cycle time and improve attrition rates in drug development.

## Competing interests

The author(s) declare that they have no competing interests.

## Supplementary Material

Additional file 1 Merck study samples. The data provided represent SVM analysis results on Merck study. Click here for file
