# Supplementary material for: Diagnosis of drug-induced renal tubular toxicity using global gene expression profiles
Source: J Transl Med. 2007 Oct 1;5:47. doi: 10.1186/1479-5876-5-47 (PMC2194664; doi:10.1186/1479-5876-5-47)
Supplement: Additional file 1 — Merck study samples. The data provided represent SVM analysis results on Merck study. [file 1479-5876-5-47-S1.doc]

**Additional files**

Additional file 1

File format: DOC

Title: Merck study samples

Description: The data provided represent SVM analysis results on Merck study.

Merck samples with histopathological grade and binary class label for SVM classification. The columns are: A_id, the animal identification number; C.Dose.Day, Compound.Dose.Day; H_score, histopathology grade; B_class, the designated SVM class label for binary classification training or testing. The compounds are: Cis (Cisplatin), Cyclo (Cyclosporine), Gent (Gentamycin), Merck compound X, NaF and Veh (Vehicle control). The binary class label was assigned based on the pathology grade. 1-5 labeled as 1, the positive class and 0 labeled as -1, the negative class.

| A_id | C.Dose.Day | H_score | B_class |  | A_id | C.Dose.Day | H_score | B_class |
| --- | --- | --- | --- | --- | --- | --- | --- | --- |
| 1 | Cis.000.03 | 0 | -1 |  | 106 | Gent.240.03 | 1 | 1 |
| 2 | Cis.000.03 | 0 | -1 |  | 107 | Gent.240.03 | 0 | -1 |
| 3 | Cis.000.03 | 0 | -1 |  | 108 | Gent.240.03 | 0 | -1 |
| 4 | Cis.000.03 | 0 | -1 |  | 109 | Gent.240.03 | 0 | -1 |
| 5 | Cis.000.03 | 0 | -1 |  | 110 | Gent.240.09 | 4 | 1 |
| 6 | Cis.000.08 | 0 | -1 |  | 111 | Gent.240.09 | 4 | 1 |
| 7 | Cis.000.08 | 0 | -1 |  | 112 | Gent.240.09 | 4 | 1 |
| 8 | Cis.000.08 | 0 | -1 |  | 113 | Gent.240.09 | 3 | 1 |
| 9 | Cis.000.08 | 0 | -1 |  | 114 | Gent.240.12 | 5 | 1 |
| 10 | Cis.005.03 | 0 | -1 |  | 115 | Gent.240.12 | 5 | 1 |
| 11 | Cis.005.03 | 0 | -1 |  | 116 | Gent.240.12 | 5 | 1 |
| 12 | Cis.005.03 | 0 | -1 |  | 117 | Gent.240.12 | 5 | 1 |
| 13 | Cis.005.03 | 0 | -1 |  | 118 | X.000.03 | 0 | -1 |
| 14 | Cis.005.08 | 0 | -1 |  | 119 | X.000.03 | 0 | -1 |
| 15 | Cis.005.08 | 0 | -1 |  | 120 | X.000.03 | 0 | -1 |
| 16 | Cis.005.08 | 0 | -1 |  | 121 | X.000.03 | 0 | -1 |
| 17 | Cis.005.08 | 0 | -1 |  | 122 | X.000.08 | 0 | -1 |
| 18 | Cis.035.03 | 2 | 1 |  | 123 | X.000.08 | 0 | -1 |
| 19 | Cis.035.03 | 2 | 1 |  | 124 | X.000.08 | 0 | -1 |
| 20 | Cis.035.03 | 2 | 1 |  | 125 | X.000.08 | 0 | -1 |
| 21 | Cis.035.03 | 2 | 1 |  | 126 | X.000.14 | 0 | -1 |
| 22 | Cis.035.08 | 4 | 1 |  | 127 | X.000.14 | 0 | -1 |
| 23 | Cis.035.08 | 4 | 1 |  | 128 | X.000.14 | 0 | -1 |
| 24 | Cis.035.08 | 4 | 1 |  | 129 | X.000.14 | 0 | -1 |
| 25 | Cis.035.08 | 4 | 1 |  | 130 | X.075.03 | 1 | 1 |
| 26 | Cis.070.03 | 2 | 1 |  | 131 | X.075.03 | 0 | -1 |
| 27 | Cis.070.03 | 2 | 1 |  | 132 | X.075.03 | 0 | -1 |
| 28 | Cis.070.03 | 2 | 1 |  | 133 | X.075.03 | 0 | -1 |
| 29 | Cis.070.03 | 2 | 1 |  | 134 | X.075.03 | 0 | -1 |
| 30 | Cis.070.08 | 5 | 1 |  | 135 | X.075.08 | 0 | -1 |
| 31 | Cis.070.08 | 5 | 1 |  | 136 | X.075.08 | 0 | -1 |
| 32 | Cis.070.08 | 5 | 1 |  | 137 | X.075.08 | 0 | -1 |
| 33 | Cis.070.08 | 5 | 1 |  | 138 | X.075.08 | 0 | -1 |
| 34 | Cyclo.06.03 | 0 | -1 |  | 139 | X.075.08 | 0 | -1 |
| 35 | Cyclo.06.03 | 0 | -1 |  | 140 | X.075.14 | 0 | -1 |
| 36 | Cyclo.06.03 | 0 | -1 |  | 141 | X.075.14 | 0 | -1 |
| 37 | Cyclo.06.03 | 0 | -1 |  | 142 | X.075.14 | 0 | -1 |
| 38 | Cyclo.06.09 | 0 | -1 |  | 143 | X.075.14 | 0 | -1 |
| 39 | Cyclo.06.09 | 0 | -1 |  | 144 | X.075.14 | 0 | -1 |
| 40 | Cyclo.06.09 | 0 | -1 |  | 145 | X.150.03 | 2 | 1 |
| 41 | Cyclo.06.09 | 0 | -1 |  | 146 | X.150.03 | 2 | 1 |
| 42 | Cyclo.06.15 | 0 | -1 |  | 147 | X.150.03 | 2 | 1 |
| 43 | Cyclo.06.15 | 0 | -1 |  | 148 | X.150.03 | 2 | 1 |
| 44 | Cyclo.06.15 | 0 | -1 |  | 149 | X.150.03 | 2 | 1 |
| 45 | Cyclo.06.15 | 0 | -1 |  | 150 | X.150.08 | 3 | 1 |
| 46 | Cyclo.30.03 | 0 | -1 |  | 151 | X.150.08 | 3 | 1 |
| 47 | Cyclo.30.03 | 0 | -1 |  | 152 | X.150.08 | 3 | 1 |
| 48 | Cyclo.30.03 | 0 | -1 |  | 153 | X.150.08 | 2 | 1 |
| 49 | Cyclo.30.03 | 0 | -1 |  | 154 | X.150.08 | 2 | 1 |
| 50 | Cyclo.30.09 | 0 | -1 |  | 155 | X.150.14 | 1 | 1 |
| 51 | Cyclo.30.09 | 0 | -1 |  | 156 | X.150.14 | 1 | 1 |
| 52 | Cyclo.30.09 | 0 | -1 |  | 157 | X.150.14 | 1 | 1 |
| 53 | Cyclo.30.09 | 0 | -1 |  | 158 | X.150.14 | 1 | 1 |
| 54 | Cyclo.30.15 | 2 | 1 |  | 159 | X.150.14 | 1 | 1 |
| 55 | Cyclo.30.15 | 1 | 1 |  | 160 | X.225.03 | 3 | 1 |
| 56 | Cyclo.30.15 | 1 | 1 |  | 161 | X.225.03 | 3 | 1 |
| 57 | Cyclo.30.15 | 0 | -1 |  | 162 | X.225.03 | 3 | 1 |
| 58 | Cyclo.60.03 | 0 | -1 |  | 163 | X.225.08 | 3 | 1 |
| 59 | Cyclo.60.03 | 0 | -1 |  | 164 | X.225.08 | 3 | 1 |
| 60 | Cyclo.60.03 | 0 | -1 |  | 165 | X.225.08 | 3 | 1 |
| 61 | Cyclo.60.03 | 0 | -1 |  | 166 | NaF.00.03 | 0 | -1 |
| 62 | Cyclo.60.09 | 1 | 1 |  | 167 | NaF.00.03 | 0 | -1 |
| 63 | Cyclo.60.09 | 1 | 1 |  | 168 | NaF.00.03 | 0 | -1 |
| 64 | Cyclo.60.09 | 1 | 1 |  | 169 | NaF.00.03 | 0 | -1 |
| 65 | Cyclo.60.09 | 0 | -1 |  | 170 | NaF.00.03 | 0 | -1 |
| 66 | Cyclo.60.15 | 2 | 1 |  | 171 | NaF.00.08 | 0 | -1 |
| 67 | Cyclo.60.15 | 2 | 1 |  | 172 | NaF.00.08 | 0 | -1 |
| 68 | Cyclo.60.15 | 2 | 1 |  | 173 | NaF.00.08 | 0 | -1 |
| 69 | Cyclo.60.15 | 1 | 1 |  | 174 | NaF.00.08 | 0 | -1 |
| 70 | Gent.000.03 | 0 | -1 |  | 175 | NaF.00.08 | 0 | -1 |
| 71 | Gent.000.03 | 0 | -1 |  | 176 | NaF.00.12 | 0 | -1 |
| 72 | Gent.000.03 | 0 | -1 |  | 177 | NaF.00.12 | 0 | -1 |
| 73 | Gent.000.03 | 0 | -1 |  | 178 | NaF.00.12 | 0 | -1 |
| 74 | Gent.000.09 | 0 | -1 |  | 179 | NaF.00.12 | 0 | -1 |
| 75 | Gent.000.09 | 0 | -1 |  | 180 | NaF.00.12 | 0 | -1 |
| 76 | Gent.000.09 | 0 | -1 |  | 181 | NaF.35.03 | 1 | 1 |
| 77 | Gent.000.09 | 0 | -1 |  | 182 | NaF.35.03 | 1 | 1 |
| 78 | Gent.000.15 | 0 | -1 |  | 183 | NaF.35.03 | 1 | 1 |
| 79 | Gent.000.15 | 0 | -1 |  | 184 | NaF.35.03 | 1 | 1 |
| 80 | Gent.000.15 | 0 | -1 |  | 185 | NaF.35.03 | 1 | 1 |
| 81 | Gent.000.15 | 0 | -1 |  | 186 | NaF.35.08 | 2 | 1 |
| 82 | Gent.020.03 | 0 | -1 |  | 187 | NaF.35.08 | 2 | 1 |
| 83 | Gent.020.03 | 0 | -1 |  | 188 | NaF.35.08 | 2 | 1 |
| 84 | Gent.020.03 | 0 | -1 |  | 189 | NaF.35.08 | 2 | 1 |
| 85 | Gent.020.03 | 0 | -1 |  | 190 | NaF.35.08 | 1 | 1 |
| 86 | Gent.020.09 | 0 | -1 |  | 191 | NaF.35.12 | 2 | 1 |
| 87 | Gent.020.09 | 0 | -1 |  | 192 | NaF.35.12 | 2 | 1 |
| 88 | Gent.020.09 | 0 | -1 |  | 193 | NaF.35.12 | 2 | 1 |
| 89 | Gent.020.09 | 0 | -1 |  | 194 | NaF.35.12 | 2 | 1 |
| 90 | Gent.020.15 | 1 | 1 |  | 195 | NaF.35.12 | 1 | 1 |
| 91 | Gent.020.15 | 1 | 1 |  | 196 | NaF.75.03 | 3 | 1 |
| 92 | Gent.020.15 | 0 | -1 |  | 197 | NaF.75.03 | 2 | 1 |
| 93 | Gent.020.15 | 0 | -1 |  | 198 | NaF.75.03 | 2 | 1 |
| 94 | Gent.080.03 | 1 | 1 |  | 199 | NaF.75.03 | 1 | 1 |
| 95 | Gent.080.03 | 0 | -1 |  | 200 | NaF.75.08 | 3 | 1 |
| 96 | Gent.080.03 | 0 | -1 |  | 201 | NaF.75.08 | 2 | 1 |
| 97 | Gent.080.03 | 0 | -1 |  | 202 | NaF.75.08 | 2 | 1 |
| 98 | Gent.080.09 | 2 | 1 |  | 203 | NaF.75.08 | 2 | 1 |
| 99 | Gent.080.09 | 2 | 1 |  | 204 | NaF.75.08 | 2 | 1 |
| 100 | Gent.080.09 | 1 | 1 |  | 205 | NaF.75.12 | 3 | 1 |
| 101 | Gent.080.09 | 0 | -1 |  | 206 | NaF.75.12 | 3 | 1 |
| 102 | Gent.080.15 | 2 | 1 |  | 207 | NaF.75.12 | 3 | 1 |
| 103 | Gent.080.15 | 2 | 1 |  | 208 | NaF.75.12 | 2 | 1 |
| 104 | Gent.080.15 | 2 | 1 |  | 209 | NaF.75.12 | 2 | 1 |
| 105 | Gent.080.15 | 2 | 1 |  |  |  |  |  |
